# Initial dosimetric evaluation of SmartArc – a novel VMAT treatment planning module implemented in a multi‐vendor delivery chain

**DOI:** 10.1120/jacmp.v11i1.3169

**Published:** 2010-01-28

**Authors:** Vladimir Feygelman, Geoffrey Zhang, Craig Stevens

**Affiliations:** ^1^ Division of Radiation Oncology H. Lee Moffitt Cancer Center Tampa Florida 33612 USA

**Keywords:** VMAT, IMRT, treatment planning system commissioning, dose‐error analysis, diode array dosimeter

## Abstract

We performed an initial dosimetric evaluation of SmartArc – a novel VMAT planning module for the Philips Pinnacle treatment planning system. It was implemented in a multi‐vendor environment, with the other two major components of the delivery chain being MOSAIQ record and verify system (IMPAC Medical Systems, Sunnyvale, CA) and a Trilogy linac (Varian Medical Systems, Palo Alto, CA). A test suite of structure sets and dose objectives provided by the AAPM for multi‐institutional comparison of IMRT dosimetry was used. A total of fifty plans were successfully delivered. The effect of control point spacing on dosimetric accuracy was investigated. When calculated with the 4° spacing, the overall mean point dose errors measured with an ion chamber were 0.5±1.4and −0.3±1.4% for the PTV and OAR, respectively. The γ(3%, 3 mm) passing rate, measured for absolute dose with a biplanar diode array, was 98.2±1.6% (range 94.5–99.9%). Ninety percent of the passing rate values were above 97.7%. With the 6° control point spacing, the highly modulated plans exhibited large dosimetric errors (e.g. γ(3%, 3 mm) passing rates below 90% and ion chamber point dose errors of 6–12%), while the results were still acceptable for the simpler cases. The data show that the practical accuracy of the small‐arc approximation, which is at the heart of VMAT dose calculations, depends not only on the control point spacing, but also on the size and relative position of the MLC openings corresponding to the consecutive control points. The effect of the minimum allowed separation between the opposing leaves was found to be minimal. It appears that 4° control point spacing may be a good compromise between calculation speed and accuracy. However each institution is encouraged to establish its own treatment planning guidelines based on the case complexity and acceptable error level.

PACS number: 87.55Qr

## I. INTRODUCTION

Volumetric modulated arc therapy (VMAT) offers potential dosimetric and efficiency advantages by being able to deliver modulated cone‐beam radiation from a single or multiple arc(s).^(^ ^1^ ^,^ ^2^ ^)^ This technique is characterized by the simultaneous rotation of the gantry and movement of the multileaf collimator (MLC). The gantry speed and dose rate can vary, differentiating the VMAT delivery from the previously described intensity‐modulated arc therapy (IMAT).^(2)^ In the latter, the fluence rate and gantry speed are kept constant. The comprehensive theory of the VMAT delivery scheme is yet to be developed.^(3)^ However, two commercial solutions were successfully introduced, and it was demonstrated that both RapidArc from Varian (Varian Medical Systems, Palo Alto, CA) and VMAT (generic term adopted by the manufacturer) from Elekta (Elekta Ltd, Crawley, UK) can be commissioned with the dosimetric accuracy that compares favorably with other methods of IMRT delivery.^(^ ^4^ ^–^ ^6^ ^)^ In particular, Ling et al.^(6)^ demonstrated that the MLC movement, variable dose rates, and gantry speeds of a Varian accelerator can be controlled precisely for accurate RapidArc delivery. Korremann et al.^(5)^ reported good agreement between measured and calculated dose distributions for RapidArc with gamma (3%, 3 mm) values below 1 for more than 95% of the measurement points. Similar results were reported by Bedford et al.^(4)^ for VMAT delivery on an Elekta accelerator.

In this paper, we provide the results of the initial evaluation of the prototype commercial treatment planning module designed to support VMAT delivery in a variety of multi‐vendor configurations. This module called SmartArc is available in Pinnacle treatment planning system (TPS) v.9 (Philips Radiation Oncology Systems, Fitchburg, WI). The specific multi‐vendor delivery chain studied in this work included a Varian linac and a MOSAIQ record and verify system (IMPAC Medical Systems Inc., Sunnyvale, CA). The major goal of the paper is to systematically asses the difference between calculated and measured dose, using the standardized set of contours and dose‐volume objectives. In the process, we study the effects of varying some important user‐selectable parameters, such as angular spacing of control points and maximum allowed delivery time.

## II. MATERIALS AND METHODS

### A. User‐selectable SmartArc parameters

A description of the optimization algorithm was published by Bzdusek et al.^(7)^ It is, at least in principle, designed to support any linear accelerator that can deliver dynamic arcs. At first, a coarse set of segments spaced 24° around the user‐selected arc is generated. An initial intensity modulation optimization is performed. The resulting fluence maps are converted into segments, which are then filtered, redistributed, and interpolated to achieve the desired fine angular distribution around the arc. Available options for the final control point spacing are 6, 4, 3 or 2°. The segments are optimized to satisfy the dose‐volume objectives and delivery constraints. Finally, the full convolution calculation is performed followed by additional segment weight optimization.

The algorithm largely uses the machine and beam models from the current version of Pinnacle,^(8)^ although some additional parameters need to be specified to explicitly describe the constraints of the dynamic delivery.^(9)^ The algorithm has a number of optimization modes in terms of varying the gantry speed and dose rate. Assuming a Varian linac fully licensed for VMAT, the dose rate is set to continuously variable. The most interesting option is “Limit gantry acceleration?”. When set to “Yes”, the optimizer takes full control of the delivery time, the gantry, and leaf motion parameters, and user intervention in terms of the delivery constraints is not required. The delivery time is expected to follow closely the Pinnacle estimate. The maximum gantry acceleration/deceleration per control point has to be specified in the machine definition tab, with the recommended value being 0.75 cm/sec per control point.^(9)^ When “Limit gantry acceleration?” is set to “No”, the plans are calculated with the constant gantry speed. The user specifies the maximum desired delivery time. At the same time, the user must enter the leaf motion constraint in terms of cm per degree to assure continuous delivery (the so called “efficiency constraint”^(1)^). With the user‐selectable maximum gantry speed set at 4.8 deg/sec (75 sec full rotation period) and the maximum leaf speed at 2.25 cm/sec, as recommended by Philips,^(9)^ the leaf motion may not exceed 0.469 cm/deg. Once the plan is transferred to the linac, its software optimizes the gantry speed and dose rate for the fastest possible delivery. Both the gantry speed and dose rate vary and, as a result, the actual delivery time can differ substantially from the estimated value, which assumes a constant gantry speed. For this work, we chose to use this option because it affords more flexibility for research. We also decided to keep the maximum leaf speed at the vendor‐recommended 2.25 cm/sec, although it was successfully tested for a similar accelerator at 2.86 cm/sec.^(6)^

Among the parameters common to all optimization modes, maximum monitor unit per degree was set to 20 MU/deg for the standard beams (600 MU/min maximum dose rate). The maximum number of MU for Varian VMAT standard X‐ray beams has a hard limit of 999. The accelerator dose rate table is now a part of the machine definition in Pinnacle because the VMAT plans explicitly incorporate the dose rate into the optimization process.

From the planner's point of view, a few additional parameters also need to be defined prior to optimization. One or two full or partial arcs per SmartArc beam are allowed. We use the term “beam” for two arcs in a sense that both of them rely on each other and are optimized simultaneously. Couch angulations are permitted, although no anticollision rules are enforced by the software and it is up to the operator to determine if the plan can be delivered safely. The minimum separation between opposing MLC leaves was available as a user‐selectable parameter in the test version. The rest of the planning process is similar to the current one in Pinnacle, except that hard dose constraints are not allowed and the dose‐volume objectives can be modified without stopping the optimization process.

### B. Delivery chain components

We used a Varian Trilogy linac with 6 and 15 MV X‐ray energies, Millennium 120‐leaf MLC, and 4D treatment console (4DTC) software v.8. The software was upgraded with the VMAT capability. Quality assurance program for the machine includes the components critical for the VMAT treatments such as MLC picket fence tests at different gantry angles, dynalog file analysis for dynamic arcs, and output constancy with varying dose rate. It was demonstrated^(6)^ that when a Varian linac performs within specifications, it is capable of synchronization of the gantry angle, dose rate, and MLC position necessary for the accurate delivery of the VMAT treatment. For a number of delivered beams, we recorded the dynalog results from both the accelerator console (MU and gantry angle RMS deviations) and the 4DTC (leaf position deviations).

Treatment plans were transferred via DICOM RT from the TPS to the record and verify (R&V) system – MOSAIQ v. 1.6. It is important to ensure that in the R&V machine characterization file the IMAT (gantry indexed) option is set to “No” and the VMAT (MU indexed) to “Yes”. In this configuration, the essential plan information that is transferred from the TPS to the accelerator through the R&V system is the total MU number, maximum dose rate and MU increment per control point (CP). It is left up to the accelerator software to determine the exact gantry and leaf speeds and the dose rate to deliver the plan in the shortest period of time. We studied the empirical relationship between the estimated and actual delivery time as a function of the user‐defined maximum allowed delivery time. This is important because speedy delivery is one of the major benefits of the VMAT technique.

### C. Planning

**Figure 1:**
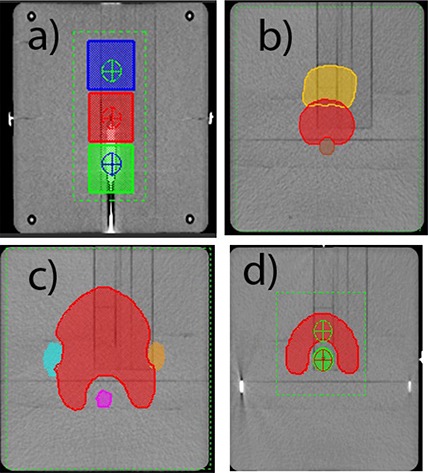
Planning contours projected on the Cube phantom: (a) stacked cylinders – coronal view through isocenter, (b)mock prostate – transverse 2.3 cm superior, (c) mock H&N – transverse 3.5 cm superior, and (d) C‐shape – transverse through isocenter.

We used a suite of IMRT commissioning structure sets (Fig. 1), dose objectives, and measurement specifications available from the AAPM website (http://www.aapm.org/pubs/tg119/default.asp). This suite was developed by the AAPM Task Group 119 to assist with IMRT commissioning process. Using these datasets, as opposed to an arbitrary collection of local cases, should facilitate meaningful comparison between different institutions, particularly with those participating in the task group, once the final report is published. We adhered as closely as possible to the document specifications, with the notable exception of substituting 360° arcs for the seven‐ or nine‐beam step and shoot IMRT arrangements. Four sets of structures in the suite were used: stacked cylinders, mock prostate, mock head and neck, and a C‐shaped structure with an avoidance core, representative of a perispinal case. All dose objectives summarized in Table 1 are specified as points on the dose‐volume histogram (DVH). For example, notation $D_{10}=55\text{Gy}$ refers to the point at the intersection of 55 Gy on the dose axis and 10% of the structure volume on the (normalized) volume axis. A total of five sets of plans were generated, as the C‐shape structure set had two sets of objectives – the easier and the harder one. The latter are not expected to be achieved with the typical linac technology. The structure sets were imported via DICOM RT and registered at the center of the Plastic Water $20\times 20\times 20\;{\text{cm}}^{3}$ Cube phantom (CIRS Inc., Norfolk, VA).

**Table 1:** Dose objectives and ion chamber measurement points. For optimization, all plans used full 360 arc(s) with a 6 MV beam.

*Plan*	*Structure*	*Prescribed Dose, Gy*	*Dose Limit, Gy*	*Measurement Point in IEC Coordinates, cm*
Multi‐target (three 4 cm long, 4 cm diameter cylinders)	Central cylinder	$D_{10}\geq 50$	$D_{10}\leq 53$	X0,Y0,Z0
	Superior cylinder	$D_{99}\leq 25$	$D_{10}\leq 35$	X0,Y4,Z0
	Inferior cylinder	$D_{99}\geq 1250$	$D_{10}\leq 25$	X0,Y‐4,Z0
Mock Prostate	PTV	$D_{95}\geq 75.6$	$D_{5}\leq 83$	X0,Y0,Z0
	Rectum		$D_{30}\leq 70$; $D_{10}\leq 75$	X0,Y0,Z‐2.5
	Bladder		$D_{30}\leq 70$; $D_{10}\leq 75$
Mock H&N	PTV	$D_{99}\geq 46.5$	$D_{20}\leq 55$	X0,Y0,Z0
		$D_{90}\geq 50$
	Cord		Max 40	X0,Y0,Z‐3.5
	Parotids		D50≤20
C‐shape	PTV	$D_{95}\geq 50$	$D_{10}\leq 55$	X0,Y0,Z2.5
(easier)	Core (OAR)		$D_{5}\leq 25$	X0,Y0,Z0
C‐shape	PTV	$D_{95}\geq 50$	$D_{10}\leq 55$	X0,Y0,Z2.5
(harder)	Core (OAR)		$D_{5}\leq 10$	X0,Y0,Z0

Single‐arc plans were used whenever possible. Double‐arc plans were explored when objectives were hard to meet. All the plans were calculated with the adaptive version of the collapsed cone convolution algorithm^(10)^ on a 3 mm grid. Five highly modulated plans were recalculated on the 2 mm and one on the 1 mm grid for comparison. It was verified that the interpolation error was negligible in a homogeneous phantom. All the plans used the dose objectives from Table 1 as SmartArc optimization parameters. After the final dose calculation was performed, an additional separate segment weight optimization routine was invoked.

To study the effect of the maximum allowed delivery time on the plan quality, dosimetric agreement and actual delivery time for each site a series of plans with different maximum delivery times were developed. The time limit was incrementally increased from the minimal allowed (approximately 75 sec) up to the point when the estimated time became less than the specified maximum time. To directly compare the quality of these plans, the total MUs were adjusted slightly to obtain the same target coverage on the DVH.

The next set of tests evaluated the effect of varying the angular CP spacing on the plan quality and dosimetric agreement. It was postulated^(7)^ that a 4° gantry angle interval between the MLC CPs may be a good compromise between the speed and accuracy of the dose calculations. The majority of the plans were optimized with this setting, although at least one plan for each structure set was optimized with the 2° and 6° CP spacing to study the effect of discretization on dosimetric agreement. Secondary to that, the influence of the CP spacing on the planning time was studied. To that end, the calculation times for two plans – one simpler (prostate) and one more complex (C‐shape) were recorded for the CP spacing of 2, 4, and 6°. The times for each stage of the calculation – variable registration and initial fluence optimization, intermediate convolution calculation, machine parameter optimization, and final dose calculation – were obtained. The number of iterations was set high enough so that in each case the optimization process terminated on convergence of the objective function.

Finally, the effect of the minimum leaf separation on dosimetric agreement was evaluated on a suitable subset of plans.

Most of the work was performed with the 6 MV beam. A number of plans were recalculated (without reoptimization) for the 15 MV beam, to access the agreement between the measured and calculated dose for a high energy beam.

### D. Verification

#### D.1 Ion chamber measurements

The Cube phantom can accommodate a cylindrical PTW TN 31015 PinPoint chamber (PTW‐Freiburg, Germany) in a movable insert. The small volume of the chamber (0.03 cm^3^) allows for point dose comparison in the PTV and organs at risk (OAR), obviating the need to determine the average active volume dose from the DVH. After irradiating the chamber to a few hundred MUs and zeroing of the PTW Unidos‐E electrometer, the residual leakage was negligible. The accelerator output variation was accounted for by establishing the reading per MU for a $10\times 10\;{\text{cm}}^{2}$ field at 10 cm depth at isocenter. Following the TG119 guidelines, dose deviations were expressed as a percentage of the prescription dose. Measurement points are specified in Table 1.

#### D.2 Diode array measurements

Absolute dose distributions were measured with a biplanar diode array (${\text{Delta}}^{4}$, ScandiDos AB, Uppsala, Sweden). This device was previously validated for IMRT^(^ ^11^ ^–^ ^13^ ^)^ and VMAT^(12)^ dosimetry. It was used for commissioning of the Varian^(5)^ and Elekta^(4)^ VMAT systems, with good results. It has 1069 diodes arranged on two orthogonal planes situated in a cylindrical PMMA phantom 22 cm in diameter. The active area is $20\times 20\;{\text{cm}}^{2}$ in each plane. The diodes are spaced 5 mm apart in the central $6\times 6\;{\text{cm}}^{2}$ area and 10 mm apart elsewhere on the plane. The diode arrays undergo relative and absolute calibration, and the readings for each detector are corrected for temperature, filed size, depth, and angular response. The measurement cycle is triggered by the accelerator synchronization pulse, and the gantry angle is acquired by an independent inclinometer. The accelerator output variation is taken into account by a daily correction factor derived from measuring a simple reference plan consisting of the two opposed lateral $10\times 10\;{\text{cm}}^{2}$ beams. For IMRT verification, the plan in question is projected on the ${\text{Delta}}^{4}$ phantom and recalculated. The monitor units are kept the same as in the original plan. The resulting reference dose is transferred to the ${\text{Delta}}^{4}$ software along with the plan information as DICOM RT objects. It is then compared with the measurement in the ${\text{Delta}}^{4}$ software using standard dose‐comparison tools. Since the ${\text{Delta}}^{4}$ phantom is similar in size to the Cube phantom, the dose deviation was again expressed as a percentage of the prescription dose. The primary method of comparing the measured and calculated dose in this work was gamma analysis^(14)^ with the 3% (absolute) dose error and 3 mm distance to agreement thresholds. Percentage of detectors with less than 3% error is also reported. Detectors receiving at least 10% of the prescription dose were included in the analysis. A highly modulated plan (C‐shape) was measured on two different occasions, demonstrate good setup reproducibility.

#### D.3 Couch attenuation

Although the Varian IGRT couch is designed to minimize beam attenuation for kV imaging, it still introduces some attenuation in the megavoltage beams, especially 6 MV. Vanetti et al.^(15)^ estimated the maximum dose error for RapidArc prostate plans not taking the couch attenuation into account at 2.1% in the PTV. Pinnacle currently does not correct the calculated dose for treatment couch attenuation. A simple empirical method was used instead. A Farmer ion chamber with a buildup cap was positioned at isocenter 15 cm above the table surface. A full arc with a $10\times 10\;{\text{cm}}^{2}$ field was delivered first with the table in the beam and then with the chamber suspended from the end of the couch. The ratio of the chamber readings was taken to approximate the couch attenuation for a 360° arc. Both ion chamber and ${\text{Delta}}^{4}$ measured doses were adjusted accordingly. The table longitudinal position used for the first arc (couch in the beam) was recorded and used subsequently for all ion chamber and ${\text{Delta}}^{4}$ measurements, since the couch thickness varies along its length.^(15)^

#### D.4 General

Statistical analysis was performed with GraphPad Prism v.5 software (GraphPad Software, San Diego CA). Average leaf pair opening (ALPO)^(16)^ for each arc was calculated by RadCalc v. 5.2 (LifeLine Software Inc., Tyler, TX). ALPO is calculated as a weighted average of all non‐zero leaf pair openings for all control points. The weight assigned to each control point is proportional to the number of MUs. It provides an indication of the aperture sizes used by the optimization algorithm.

## III. RESULTS

### A. Dynalog results

A total of fifty SmartArc plans were delivered at least twice without any errors on the 4D treatment console or linac interlocks. Twelve plans of varying complexity had the associated dynalog files examined. On the linac side, the reported average angle and MU errors were 0.30±0.04° and 0.05±0.01MU, respectively. For the MLC dynalog files, the reported RMS average and maximum values averaged across the twelve plans were 0.049±0.009and 0.06±0.01cm, respectively. For the two plans the maximum error was in the 0.25–0.3 cm range, while for the remaining ten it did not exceed 0.2 cm. No pattern of difference in dynalog results for the different plans was observed.

### B. Delivery time

**Figure 2:**
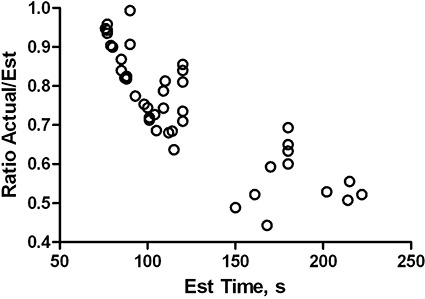
Ratio of actual to estimated delivery times as a function of estimated time.

The estimated delivery time was always at or below the user‐defined maximum delivery time. Although the ratio of the actual to estimated delivery time varies from plan to plan, it can be seen from the empirical data (Fig. 2) that the actual time is fairly close to the estimated one for the fast deliveries (75 sec) but is reduced to roughly half of the estimate for the longer delivery times.

### C. Couch attenuation

For the 6MV 360° arc beams, the ratio of the ion chamber reading with and without couch was 0.991. For 15 MV X‐rays it was 0.996. These values were used to correct both measured ion chamber point doses and ${\text{Delta}}^{4}$ dose matrices. This is an approximation that does not take into account possible differences in relative weight and filed size of the portions of the SmartArc plan delivered with and without the table intercepting the beam. However the correction is small, and even a relatively large relative error in the attenuation coefficient will have little effect on the final result.

### D. Dosimetric agreement – control points spaced at 4°

The data for different cases and plans are summarized in Tables 2–5. It includes the number of arcs, set, estimated, and actual delivery times, ALPO, MU per arc, point dose errors for both PTV and OAR, γ(3%, 3mm) passing rates, and the percentage of detectors with up to 3% relative dose error.

**Table 2:** Plan parameters and dosimetric agreement, multi‐target, 4° CP spacing.

*No. of Arcs*	*Set Max Time (sec)*	*Est. Time (sec)*	*Actual Time (sec)*	*ALPO (cm)*	*MU*	*IC Dose‐diff*., *Center (PTV1), %*	*IC Dose‐diff., Superior (PTV2), %*	*IC Dose‐diff., Inferior (PTV3), %*	γ*(3%,3mm) % pass*	*% Detectors within 3% dose error*
1	60	77	72.0	2.98	322	1.0	1.9	−1.1	99.9	85.8
1	90	79	71.4	2.93	326	0.8	1.5	−0.4	99.6	85.5
1	120	101	72.0	2.94	327	0.9	1.6	−0.9	99.3	84.9
1	180	105	72.0	2.94	327	0.9	1.4	−0.8	99.9	86.2

**Table 5:** Plan parameters and dosimetric agreement, C‐shape (easier), 4° CP spacing.

*No. of Arcs*	*Set Max Time (sec)*	*Est. Time (sec)*	*Actual Time (sec)*	*ALPO (cm)*	*MU*	*IC Dose‐diff., PTV, %*	*IC Dose‐diff., OAR, %*	γ*(3%, 3mm) % pass*	*% Detectors within 3% dose error*
1	60	77	76.2	2.37	724	2.5	−1.6	99.1	68.3
1	90	90	89.4	2.2	773	3.8	−2.5	98.1	62.8
1	120	120	100.8	1.95	836	1.4	−0.3	95.2	56.8
1	180	180	114.0	1.76	961	1.1	−2.6	96.5	58.1
1	240	222	115.8	1.67	992	−2.6	−0.8	96.4	56.4
2	90+90	88+80	72.6+72.0	2.25/2.28	399+369	1.7	−0.9	99.9	74.6
2	240+240	150+168	73.2+74.4	2.06/2.31	418+393	0.9	−0.9	99.4	55.9

#### D.1 Stacked cylinders

**Figure 3:**
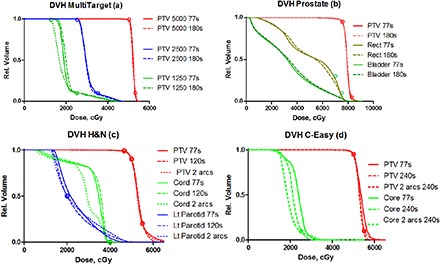
DVH curves for: (a) stacked cylinders, (b) mock prostate, (c) mock H&N, and (d) C‐shape (easier). Seconds in the legend indicate maximum delivery time set during planning. All plans have collimator angle at 15°.

The multitarget (stacked cylinders) is the first of the tests arranged according to the approximate degree of difficulty. For this simple arrangement, variation of the set maximum delivery time from 77 to 180 sec resulted in only modest increase in the estimated delivery time (Table 2. The actual delivery time and the ALPO essentially stayed the same. The DVH objectives were met regardless of the set time limit (Fig. 3(a)). The mean point dose deviation between all the plans in the central target was 0.4±0.8%. Combined mean deviation for the superior and inferior cylinders was 0.2±1.2%. Neither one was statistically different from 0 (t‐test p>0.3). The γ(3%, 3 mm) mean passing rate was 99.5±0.6%.

#### D.2 Mock prostate

This is another relatively simple case and the results are similar to the multitarget one. Allowing the algorithm more time to complete the gantry rotation did not result in the ALPO changes, indicating approximately the same degree of modulation in the beam (Table 3. The dose objectives were met with the fastest possible arc (77 sec estimated and 73 sec actual delivery time). In terms of meeting the objectives, there is essentially no difference between the DVHs for the minimum and maximum set delivery time. The mean dose deviations were 0.7±0.8and 0.2±0.3% for the PTV and OAR, respectively. The average γ(3%, 3 mm) passing percentage was 99.2±0.4%.

**Table 3:** Plan parameters and dosimetric agreement, mock prostate, 4° CP spacing.

*No. of Arcs*	*Set Max Time (sec)*	*Est. Time (sec)*	*Actual Time (sec)*	*ALPO (cm)*	*MU*	*IC Dose‐diff., PTV, %*	*IC Dose‐diff., OAR (Rectum), %*	γ*(3%,3mm) % pass*	*% Detectors within 3% dose error*
1	60	77	72.6	2.57	385	0.0	−0.2	99.6	84.1
1	90	85	73.8	2.63	382	1.4	0.1	98.8	84.4
1	120	98	73.8	2.68	375	1.4	0.6	99.3	84.5
1	180	115	73.2	2.56	392	0.0	0.2	98.9	82.5

#### D.3 Mock H&N

In this more challenging case, the algorithm begins to use all the allowed delivery time to achieve the objectives, although the actual times vary significantly less (Table 4. While the target DVHs are similar, strictly speaking only the plan with two arcs meets all the dose‐restricting objectives (Fig. 3(c)). One of the arcs uses clearly smaller apertures (ALPO 1.8 cm vs. approximately 2.8 cm for the rest of the beams). The dosimetric agreement is slightly worse than for the simpler cases. The mean point dose deviation was −1.3±1.5 and −1.7±2.4% for the PTV and cord, respectively. The average gamma passing rate was 96.1±1.8%.

**Table 4:** Plan parameters and dosimetric agreement, mock head and neck, 4° CP spacing.

*No. of Arcs*	*Set Max Time (sec)*	*Est. Time (sec)*	*Actual Time (sec)*	*ALPO (cm)*	*MU*	*IC Dose‐diff., PTV, %*	*IC Dose‐diff., OAR (Cord), %*	γ*(3%, 3mm) % pass*	*% Detectors within 3% dose error*
1	77	77	73.8	2.87	597	−2.3	−3.7	96.3	81.4
1	90	90	81.6	2.86	621	−1.4	−2.1	94.5	74.0
1	120	120	85.2	2.78	635	−2.3	−2.7	95.0	75.5
2	120+120	114+112	78.0+76.2	2.86/1.78	490+410	0.9	1.7	98.6	80.6

#### D.4 C‐shape (easier)

Fulfilling the dose objectives in this case requires an even higher degree of modulation. In this section, we are presenting the results with no limit placed on the minimum leaf separation. As the allowed delivery time increases, the optimization algorithm employs progressively smaller apertures as measured by the ALPO for the single‐arc plans (Table 5. It helps to meet the objectives, as the fastest plan with the largest ALPO is clearly inferior. The slowest single‐arc plan is very close to satisfying the objectives, but strictly speaking all of them are met only with the two‐arc plans (the DVH for one of the two‐arc plans, the last one in Table 5, is included in Fig. 3(d)). There is no apparent correlation between the ALPO and the point dose error. For all the plans in Table 5, the mean PTV and OAR dose errors were 1.3±2.0and −1.4±0.9%, respectively. The mean γ(3%, 3 mm) passing rate was 97.8±1.8%.

#### D.5 C‐shape (harder)

Based on the results from the previous section, a two‐arc plan was generated to come as close as possible to the dose objectives. As expected, fulfilling the objectives proved impossible. The solution converged to a compromise where the required target coverage was not achieved while the dose limits in both the PTV and OAR were exceeded. The point dose errors were 2.2 and −0.7% in the PTV and OAR, respectively. The gamma analysis passing rate was 95.7%.

#### D.6 Overall statistics – 6 MV

Between the four datasets in the test suite, we have calculated and measured 29 plans employing the 6 MV beam, 4° CP spacing, and no minimum gap between opposing MLC leaves. This resulted in 29 PTV and 35 OAR data points. Both the PTV and OAR point dose error distributions passed D'Augustino & Pearson normality test (p>0.3). The mean values were 0.5±1.4and −0.3±1.4% for the PTV and OAR, respectively. The data fails to demonstrate statistically significant deviation of the mean values from zero (t‐test p≥0.06). The γ(3%, 3 mm) passing rate for absolute dose was 98.2±1.6% (range 94.5–99.9%). Ninety percent of the passing rate values were above 97.7%.

#### D.7 Overall statistics – 15 MV

Seventeen plans previously generated for the 6 MV beam were recalculated without reoptimization with 15 MV X‐rays. This resulted in 17 PTV and 19 OAR measured point doses which were compared with the TPS calculations. Both PTV and OAR error distributions are consistent with normal, with the mean values of 0.5±1.4and −0.3±1.4%, respectively. Both means are not statistically different from zero (t‐test p>0.16). Point dose error analysis results for the 15MV beam are very close to the 6 MV one. Eleven plans were measured with the ${\text{Delta}}^{4}$ yielding the average γ(3%, 3 mm) passing rate of 98.9±1.4% (range 95.2–100%). Ninety percent of the passing rate values were above 98%. Again, the results are similar to the 6 MV beam.

### E. Effects of parameter variation

#### E.1 Control points spacing

**Figure 4:**
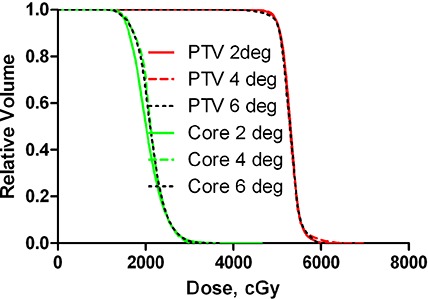
DVHs for highly modulated (C‐shape) plans optimized with 2, 4 or 6° CP spacing.

We compared dosimetric agreement for the plans optimized with the range of possible CP intervals from 6 to 2°. Figure 4 shows a set of DVHs for the highly modulated C‐shape plans optimized with the different CP spacing. The PTV DVHs are essentially indistinguishable, while there is a small difference in the OAR DVHs, with the 2° one standing apart from 4° and 6°. This is the worst case scenario, as the less complex plans would be less sensitive to the gantry angle increment. To study the discretization effect on dosimetric accuracy in isolation, it would have been ideal to have the same optimized plan with the varying number of interpolated CPs for the final calculation. In the absence of such capability, we resorted to evaluating a number of plans for different structure sets optimized with the CP spacing varying from 2 to 6°. The dose agreement results are presented in Table 6. For the first three structure sets, a representative plan previously calculated with the 4° CP spacing was reoptimized with the 2° and 6° values. For the most complex C‐shape case (easier objectives), three representative plans with varying maximum set delivery time were reoptimized and the average values are reported in Table 6.

**Table 6:** Effect of CP spacing on dosimetric agreement for different plans.

*Structure Set*	*PTV IC dose error, %*			*OAR IC dose error, %*			γ*(3%,3mm) pass, %*			*% Detectors within 3% dose error*		
*CP spacing*	*2°*	*4°*	*6°*	*2°*	*4°*	*6°*	*2°*	*4°*	*6°*	*2°*	*4°*	*6°*
Multi‐Target	0.6	0.9	1.5	−0.7	0.4	−1.9	99.9	99.3	99.6	95.5	84.9	95.5
Mock Prostate	0.3	1.4	0.9	−1.1	0.6	−0.6	99.8	99.3	100	88.8	84.5	85.6
Mock H&N	−1.0	−2.3	1.5	−0.9	−2.7	−1.5	99.5	95.0	93.8	89.5	75.5	72.2
C‐shape (easier)	0.1	0.0	**7.1**	−0.3	−1.2	**11.0**	98.3	96.0	**86.2**	68.0	57.1	51.6

Sample treatment times associated with two different plans optimized with the CP spacing varying from 2 to 6° are presented in Table 7. There is an almost exactly linear relationship between the intermediate and final calculation times and the number of control points. The same relationship holds for the overall time for the prostate plan. In the C‐shape case, the overall time slightly increases from 4 to 6°. This is due to the increased number of iteration it took the optimizer to reach the solution (Table 7.

**Table 7:** Treatment planning times (mm:ss) for single‐arc plans with different CP spacing.

*Case*	*No. Iterations*			*Fluence calc*.			*Intermediate calc*.			*Optimization*			*Final calc*.			***Total***		
	*2°*	*4°*	*6°*	*2°*	*4°*	*6°*	*2°*	*4°*	*6°*	*2°*	*4°*	*6°*	*2°*	*4°*	*6°*	***2°***	*4°*	*6°*
Prostate	37	45	41	00:24	00:20	00:21	10:02	04:50	03:17	04:11	03:02	01:36	07:58	04:01	02:31	**22:35**	**12:13**	**07:45**
C‐shape	59	64	116	00:28	00:24	00:19	07:26	03:23	02:08	09:42	05:05	07:53	05:54	02:46	01:45	**23:30**	**11:38**	**12:05**

#### E.2 Minimum leaf separation

**Figure 5:**
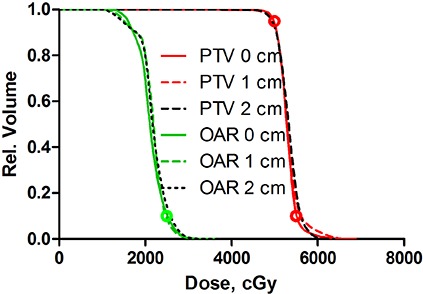
DVH comparison for C‐shape plans (easier objectives) optimized with the minimum leaf separation set to 0, 1, and 2 cm.

The effect, if any, of the minimum allowed leaf separation should be more pronounced for the highly modulated plans which tend to produce narrow segments. Therefore, it was studied for the selected C‐shape plans (easier objectives). Three plans, one single arc and two with double arcs were optimized with the minimum leaf separation set to 0, 1, or 2 cm (Table 8. The DVHs for the single arc plan are presented in Fig. 5 and the dosimetric agreement results in Table 8. Control point spacing was set at 4°.

**Table 8:** Effect of minimum leaf separation. C‐shape plans, 4° CP spacing.

*No. of Arcs*	*Set Max Time (sec)*	*Est. Time (sec)*	*Actual Time (sec)*	*Min Leaf Separation (cm)*	*ALPO*	*PTV IC dose error, %*	*OAR IC dose error, %*	γ*(3%, 3mm) % pass*	*% Detectors within 3% dose error*
1	240	222	115.8	0	1.67	−2.6	−0.8	96.4	52.5
1	240	214	108.6	1	1.92	−0.5	−0.8	96.8	53.3
1	240	170	100.8	2	2.28	−1.0	−3.3	98.4	61.5
2	90+90	88+80	72.6+72.0	0	2.25/2.28	1.7	−0.9	99.9	72.6
2	90+90	77+88	72.0+72.0	2	2.57/2.58	0.6	−0.9	99.7	70.2
2	240+240	150+168	73.2+74.4	0	2.06/2.31	0.9	−0.9	99.4	71.4
2	240+240	87+93	71.4+72	2	2.53/2.63	0.7	−0.4	99.6	73.1

## IV. DISCUSSION

### A. Dynalog results and delivery time

The dynalog results are in agreement with previously reported values.^(6)^ All the plans were delivered without any machine interlocks, which is not surprising given the fact that both the gantry speed and MLC speed limits in the SmartArc module are conservative, compared to the actual capabilities of the accelerator.

It was expected that the delivery time often would not be correctly estimated by the SmartArc algorithm^(7)^ when the planning is done with the constant gantry speed (“limit gantry acceleration” is set to “No”). Although the plan is optimized with the constant gantry speed, the VMAT‐licensed Clinac is designed to independently interpret the control point information and select the appropriate gantry speed, MLC speed, and dose rate to assure the fastest possible delivery. It was obvious to the observer that the gantry speed varied during treatment. It is understandable that there is more room to optimize the delivery speed when the gantry rotational period is not already close to its lower limit. In this work the actual delivery time never exceeded the estimate, and typically was less (Fig. 2).

### B. Dosimetric agreement, 4° CP spacing

While there is no universal prescription for the IMRT gamma analysis thresholds with electronic arrays, the survey on planar IMRT analysis^(17)^ shows that the most prevalent combination by far is 3% dose error (absolute dose) and 3 mm distance to agreement. The dose error assumes normalization to the global (typically prescription dose) value. Using these parameters with a 2D diode array, Both et al.^(18)^ demonstrated that with three different commercial TPS, 95% passing rates are reasonably achievable for the low modulated cases and 90% for more complex ones.

We have previously commissioned Pinnacle v. 8.0m for 3D and step and shoot IMRT largely incorporating the recommendations by Cadman et al.^(8)^ The model was validated for step and shoot IMRT with direct machine parameter optimization.^(19)^ For the 6 MV beam, the average point dose deviation in the PTV was 0.5±1.2% (N=17). The difference from zero was not statistically significant (t‐test p=0.1). The mean γ(3%, 3 mm) passing rate obtained with the ${\text{Delta}}^{4}$ was 97.5±2.9%.^(11)^

The emerging data on VMAT commissioning with the Delta^4^ ^(^ ^4^ ^,^ ^5^ ^,^ ^12^ ^)^ suggest that the passing rates of 95% or better could be expected. Our overall results for both 6 MV and 15 MV beams, building on the solid results with step and shoot IMRT, are in line with this expectation.

### C. Effects of parameter variation

#### C.1 Control points spacing

The most important observation here is that CP spacing is a critical user‐selectable parameter that can significantly affect the treatment planning time (Table 7 and the difference between the planned and measured doses. There is a tradeoff between calculational accuracy and speed, since the computational time increases proportionately to the number of CPs. Therefore it is preferable in practice to use the largest spacing consistent with good dosimetric results. Too coarse a step would clearly lead to diminished dosimetric accuracy. Webb and McQuaid^(3)^ formalized this concept as the “small arc approximation”, and pointed out that the approximation breaks down as the point of interest moves away from the isocenter. There is considerable variation in the literature regarding the choice of CP spacing. Otto^(1)^ postulated that to achieve accuracy consistent with the typical IMRT quality assurance criteria, 1° gantry angle sampling was necessary. Two degree separation between CPs was reported for practical planning with Varian RapidArc^(^ ^5^ ^,^ ^20^ ^)^ and 4° was postulated as optimal for the prototype of SmartArc.^(7)^ On the other hand, tomotherapy approximates full gantry rotation with 51 projections spaced just over 7° apart,^(21)^ with historically good agreement between measured and planned dose. Bedford et al.^(4)^ reported good dosimetric agreement with 10° CP spacing for relatively simple prostate and lung cases executed on an Elekta linac.

The following discussion pertains to Varian linacs used in this work. The average ${\text{Delta}}^{4}$ γ(3%, 3 mm) passing rate for all the plans optimized in this report with the 4° CP increment (98.2%) was essentially the same as 98.5% reported for RapidArc by Korreman et al.^(5)^ with 2° spacing. Both point dose and gamma analysis data for the first three structure sets in Table 6 show relatively little variation with the CP spacing. While the gamma analysis results for the H&N plan are slightly better with the smaller gantry angle increment, all the values would be considered within the clinically acceptable range (≥93.8%) even with the 6° CP spacing. The percentage of detectors with relative dose error of 3% or less is more discriminating in the H&N case, particularly when the CP spacing changes from 2 to 4° (Table 6.

The results change dramatically for the C‐shape case. Dosimetric agreement is acceptable for 2° and 4° CP spacing in terms of point‐dose errors and gamma analysis. However, both show drastic deterioration for the 6° plans. Ion chamber measurements show significant overdose in the central portion of the dose distribution. The relative dose error map from the ${\text{Delta}}^{4}$, reflected in the last column of Table 6, confirms that this is a consistent effect rather than an artifact caused by a possible ion chamber setup error in the region of high dose gradient. The relative dose error distribution broadens and skews towards the higher measured dose with the increase in CP spacing. It is clear that these errors are not caused by some beam model deficiency, such as MLC transmission or output factors for narrow elongated MLC‐defined fields. If that were the case, all similar plans would be affected approximately the same, regardless of the CP spacing.

**Figure 6:**
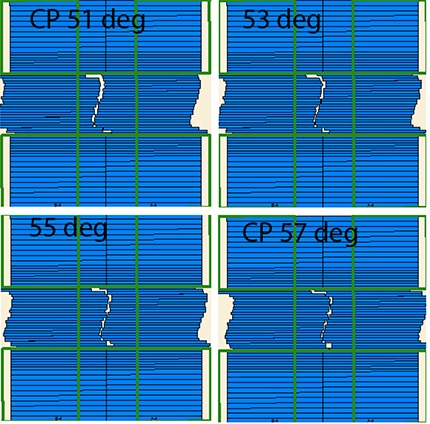
Example of the narrow MLC opening transition between control points separated by 6°. Frames of the 4D console movie are presented in two degree increments: 51 and 57° correspond to the control points, and 53 and 55° are taken in transition.

**Figure 7:**
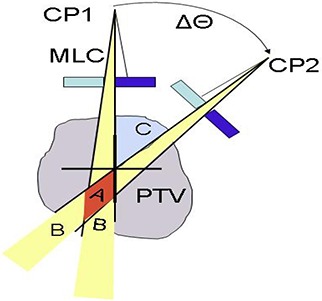
Small‐arc approximation and its limitations.

These results can be qualitatively explained based on the size and position of the beam apertures generated for the C‐shape plans. ALPOs for those plans were on the order of 1.6–2.0 cm, while they were 2.6–3.0 cm for the other structure sets. There are a substantial number of spatially separated narrow openings belonging to the consecutive CPs (see an example in Fig. 6). We can demonstrate that this would lead to the observed phenomenon of delivered dose differing from predicted. Let us assume that the gantry travels from CP1 to CP2 with the angular increment of Δθ, and the MLC leaves transition from the opening on the left of the central axis to the symmetrical opening on the right (Fig. 7). For simplicity, let us ignore the MLC transmission and penumbra. For the configuration depicted in Fig. 6, the calculated dose in area C of the PTV is zero even near the isocenter. However in reality, as the gantry angle is changing continuously, the MLC leaves are transitioning from CP1 to CP2 maintaining a gap, while monitor units are being delivered. A set of four individual representative frames from the captured screen movie of the 4D console during the delivery (Fig. 6) confirms that the leading and trailing leaves maintain approximately the same speed, and the leaves do not close between control points. As a result, area C receives non‐zero dose, contrary to the discrete calculation. This overdose area is reduced as areas B and C in Fig. 7 coalesce into A with either decreasing Δθ or increasing aperture width, particularly as it becomes more symmetrical around the central axis. In addition to depending on the CP spacing and calculation point distance to isocenter,^(3)^ our results show that the practical accuracy of the small‐arc approximation also depends on the segments’ shape and position, which is consistent with the previous observation that “the resulting dose error will be very case dependent”.^(3)^ When for the two consecutive CPs the leaves are required to travel across the central axis to produce openings on both sides, even the points at or near the isocenter can be subject to large dose errors as Δθ increases.

We designed an experimental setup, similar to Fig. 7, which further demonstrates this point by taking it to the extreme. A simple 360° plan with the constant dose rate and gantry speed was calculated with the 6° CP spacing. The control point apertures were 1 cm wide strips, centered alternatively 1.5 cm to the left and right of the central axis. This effectively created a 1 cm opening oscillating at a constant speed. The calculated dose at isocenter was 39 cGy, while the measured one was 240 cGy. When the plan was modified with inserted interpolated control points every 2°, the calculated isocenter dose increased to 198 cGy. As we see, depending on the circumstances, the dose error attributable to the CP spacing could be quite dramatic. An interesting question corollary to this is why tomotherapy reliably produces good dosimetric agreement with only 51 projections spaced 7° apart, even for highly modulated plans. The key difference between tomotherapy and linac‐based VMAT is leaf speed. In VMAT, the time it takes the leaf to travel between the CPs is by definition equal to the gantry movement time. A leaf opening resembling a shape interpolated between the CPs is maintained during the transition (Fig. 6), which is not accounted for in the dose calculation. On the other hand, the binary tomotherapy MLC reaches its position in about 20 ms, which is an order of magnitude smaller than the gantry travel time of about 200 ms or more between the projections.^(21)^ Moreover, this leaf latency effect is taken into account in the TPS.^(21)^ Therefore, tomotherapy may not be a good model for direct comparison with VMAT in terms of the dosimetric effect of CP spacing.^(3)^

#### C.2 Minimum leaf pair opening

The ALPO values in Table 8 indicate that the algorithm indeed takes advantage of the allowed narrow segments. The narrower apertures, as expected, result in the slightly better calculated DVHs (Fig. 5). The difference however is minimal. It is hard to discern any pattern in the point dose agreement data in Table 8. The gamma and relative dose‐error statistics for the double‐arc plans show no difference between 0 and 2 cm leaf spacing. There may be slightly improved dosimetric agreement with increased segment size for the single‐arc plans, but the differences are too small to draw a definitive conclusion. Based on the internal testing, the manufacturer elected to remove the minimum leaf separation as a variable parameter from the production version of SmartArc.

## V. CONCLUSIONS

Dosimetric accuracy of the Pinnacle SmartArc VMAT planning module used in conjunction with the IMPAC R&V and Varian Trilogy linac was evaluated. It is insensitive to the minimum allowed MLC leaf separation. One of the most important user‐selectable parameters is the CP spacing. Point dose errors and absolute dose gamma analysis results were acceptable for the gantry increment between the CPs of up to 4° for all the investigated plans. The most complex cases (C‐shape) exhibited large dose errors with the CP spacing of 6°, while the rest of the plans would still be considered acceptable. This indicates that the accuracy of the small‐arc approximation used in VMAT planning depends on the size and relative position of the MLC apertures. It appears that 4° CP spacing may be a good compromise between calculation speed and accuracy for the SmartArc plans delivered on Varian machines with the described user‐selectable parameters. However each institution is encouraged to establish its own guidelines based on the case complexity and acceptable error level. With the studied optimization mode, the actual delivery times correspond much closer to the maximum gantry speed than Pinnacle time estimates would suggest.

## ACKNOWLEDGEMENTS

We are thankful to Philips for providing the test version of software for evaluation, and to D. Robinson and J. Campbell for useful discussions and help with planning.
